# Depressive, anxiety, and insomnia symptoms between population in quarantine and general population during the COVID-19 pandemic: a case-controlled study

**DOI:** 10.1186/s12888-021-03108-2

**Published:** 2021-02-16

**Authors:** Chengmin Wang, Weidong Song, Xiaohui Hu, Shaoguang Yan, Xing Zhang, Xunqiang Wang, Wenli Chen

**Affiliations:** 1 Department of Psychiatry, Shenzhen Longgang Center for Chronic Disease Control, Longgang, Shenzhen, China; 2 Department of Psychiatry, Shenzhen Nanshan Center for Chronic Disease Control, Nanshan, Shenzhen, China; 3 Department of Psychiatry, Southern University of Science and Technology Hospital, Nanshan, Shenzhen, China

## Abstract

**Background:**

The COVID-19 pandemic have caused mental and psychological problems on the general population, patients, and related workers. Our study is to determine the impact of mental and psychological symptoms among population in quarantine for 2 weeks during COVID-19 pandemic.

**Methods:**

A case-controlled study design have conducted at department of psychiatry of Shenzhen Longgang Center for Chronic Disease Control in Shenzhen, China mainland from 7th April to 15th June 2020.1674 participants (aged 18 to 65 years) in quarantine for 2 weeks and 1743 age-sex matched controls living in Shenzhen were recruited between 7th April 2020 and 15th June 2020. The assessment of depressive, anxiety, and insomnia symptoms were determined by self-reported questionnaires PHQ-9, GAD-7, and ISI, respectively.

**Results:**

A total of 1674 participants in quarantine for 2 weeks and 1743 age-sex matched controls (32.6 ± 9.3 years vs. 32.7 ± 10.7 years, 49.8% vs. 47.8% females) were recruited. Population in quarantine had higher score on PHQ-9 (6.1 ± 5.5 vs. 3.0 ± 3.7, *p* < 0.001), GAD-7 (4.2 ± 4.7 vs. 1.9 ± 3.7, *p* < 0·001), and ISI (5.5 ± 5.8 vs. 3.1 ± 5.0%, *p* < 0.001) compared to general population. Population in quarantine showed significantly higher risks of depression (OR: 4.55, 95% CI: 3.82–5.41), anxiety (OR: 2.92, 95% CI: 2.43–3.51), and insomnia (OR: 2.40, 95% CI: 2.02–2.89), when compared to the general population. Younger, more education, non-married and lower household income showed higher risks of mental health problems.

**Conclusions:**

Population in quarantine had a higher level of depressive, anxiety, and insomnia symptoms than controls. Specifically, they were at a higher risk prevalence of depression, anxiety, and insomnia, especially the severity of depression, when compared to controls. Younger, more education, non-married, and lower income population in quarantine were at higher risks of mental health problems. Mental health professionals should pay attention to the mental and psychological symptoms for population in quarantine.

**Supplementary Information:**

The online version contains supplementary material available at 10.1186/s12888-021-03108-2.

## Introduction

COVID-19 disease, the infection of novel coronavirus, is a severe pneumonia pandemic. In addition to the mortality and medical consequences of patients with coronavirus infection, the COVID-19 pandemic also caused mental and psychological problems on the general population, patients, and related workers [1–3]. Under this circumstance, medical professionals and government have raised significant concerns to the mental and psychological problems of the impacted persons [1–3].

A large number of studies have shown that general population experienced a high level of stress and significant mental and psychological problems under the COVID-19 epidemic [4]. Especially those who were female, experienced negative affect, were detached, had an acquaintance infected with COVID-19, had a history of stressful situations and medical problems, showed higher levels of depression and anxiety [5]. In addition, a prospective study found that the general population still experienced a high level of stress, anxiety and depression after the peak of the COVID-19 pandemic, which suggested that the impact of the COVID-19 epidemic on the mental and psychological health of the general population might be long-lasting [6]. Otherwise, The COVID-19 patients experienced lots of mental and psychological problems, even were diagnosed with mental disorders during the whole course of COVID-19 [7]. COVID-19 might cause delirium in a significant proportion of patients in the acute stage [8]. The hospitalized patients with COVID-19 infection also experienced symptoms of depression, anxiety, insomnia and/or distress [7]. Even post-illness patients with COVID-19 infections have a higher risk of mental disorder and psychological problems [9]. Need to be noticed, there are psychological symptoms and sleep symptoms in front-line healthcare workers who participate in the fight against COVID-19 [10]. According to recent systematic review and meta-analysis, at least one in five healthcare professionals report symptoms of depression and anxiety; almost four in ten healthcare workers experience sleeping difficulties and/or insomnia. Especially, rates of anxiety and depression were higher for female healthcare workers and nursing staff, and milder mood symptoms and sub-threshold are common among healthcare workers during COVID-19 pandemic [11]. In addition, Compared with non-medical health workers, medical health workers had a higher prevalence of insomnia, anxiety, depression, somatization, and obsessive-compulsive symptoms [12]. Above all, during the COVID-19 pandemic, the prevalence of mental and psychological problems/disorders have increased among general population, patients, and healthcare workers, who being in allostatic overload were exposed to a protracted source of distress [13]. The population experienced long and continuous quarantine or isolation might be in allostatic overload status which is likely to cause more psychological distress with protracted time. Based on that*,* medical professionals and government had raised their significant concerns to mental health problems among those under quarantine.

The COVID-19 pandemic requires social distancing, quarantine and isolation, which may cause a high burden of mental and psychological problems in people without mental illness or aggravate existing conditions, especially those under quarantine [7, 14]. A recent systematic umbrella review found that patients, informal caregiver, and healthcare providers who experienced quarantine or isolation had a high burden of mental health problems, including depression, anxiety, and insomnia [15]. However, most of the included studies conducted in high-income nations and in hospital settings, then individuals and populations who have undergone quarantine and isolation were in different context [15]. In addition, based on whether they or their families /colleagues /classmates /neighbors had been quarantined, the prevalence of anxiety and depression of the affected group are higher than in the unaffected group during the COVID-19 outbreak [16].

As far as we know, there is no case-control study on the mental and psychological problems of population in quarantine and general population during COVID-19 pandemic, which highlighted the immediate impact of the isolated environment. According to recent findings focused on general population under quarantine or isolation, we hypothesized that population in quarantine had a high level of depression, anxiety, and insomnia symptoms when compared with those general population. We aimed to determine the impact of mental and psychological symptoms among population in quarantine for 2 weeks during COVID-19 pandemic by using self-reported questionnaires. These findings will identify population in quarantine for their mental and psychological symptoms during the COVID-19 pandemic and may help in implementing mental health intervention policies among those under quarantine or isolation in other countries and regions.

## Methods

### Study design and participants

The current case-controlled study was conducted from 7th April to 15th June 2020 in Shenzhen, China. Our study was submitted to and approved by The Control and Prevention Commend Office of COVID-19 Pandemic in Longgang district, Shenzhen (Document NO.: [2020]90). We recruited participants who were required to quarantine in medical observation check point for 2 weeks and community population who were lived in the same region in Shenzhen, China. Since face-to-face contact might increases the risk of infection, the study was conducted through internet-based electronic devices, including cell phone, pad, and computer. All participants provided informed consent before the mental health assessment. This study was conducted in accordance with the approved protocols and the Declaration of Helsinki.

### The inclusion criteria and exclusion criteria

The assessment was carried out after obtaining informed consent. Scan the QR code of the questionnaires with internet-based electronic devices to complete the demographic data, PHQ-9, GAD-7 and ISI among those who were quarantined for 2 weeks and general population in Shenzhen from 7th April to 15th June 2020.The other including criteria of the participants were: 1) aged from 18 to 65 years old; 2) Chinese resident (including Hong Kong) who have been required to quarantine during the COVID-19 pandemic period in Shenzhen, China; 3) no history of mental disorders, including anxiety, depression, and insomnia; 4) be able to give informed consent.

The exclusion criteria were: 1) aged younger than 18 years old or older than 65 years old; 2) without informed consent; 3) without internet-based electronic devices, including cellphone, pad, and computer; 4) unable to complete questionnaires due to severe mental or physical conditions.

### Questionnaire and data collection

Demographic characteristics of the participants, including sex, age, education, marital status, household income, and any physical or mental conditions.

Patient Health Questionnaire − 9 (PHQ-9): Depressive symptoms were assessed by the PHQ-9 which has been widely used to assess the severity of depression [17]. The PHQ-9 contains 12 items, inquiries the core and comorbid symptoms of depression during the past 2 weeks by a 4-point scale with a total score ranging from 0 to 27. The higher the score suggests higher level of depression severity. In the general Chinese population, the Chinese version of the PHQ-9 is a valid and efficient tool for screening depression, with a recommended cutoff score of 7 or more [18]. Moderate-severe depression is defined as the score of 10 or more.

Generalized Anxiety Scale (GAD-7): Anxiety symptoms were measured by the GAD-7, which was comprised of 7 items evaluating various symptoms of anxiety in the past 2 weeks [19]. Each item of the scale ranges from 0 to 3 points, with a total score of 21 points. The higher the score suggests higher level of anxiety severity. The GAD-7 had acceptable properties for identifying GAD at cutoff scores 7 [20]. Moderate-severe anxiety is defined as the score of 10 or more.

Insomnia Severity Index (ISI): The ISI was used to assess insomnia symptoms of the participants. ISI is a seven-item questionnaire evaluating the subtype, severity, and impacts of sleep difficulties in the past 2 weeks. The participants are rated on a 5-point Likert scale from 0 (not at all) to 4 (extremely), with a total score of 0 to 28 points [21]. The higher the score, the higher the level of insomnia. A cutoff of ≥8 on the ISI were found to correctly identify individuals with the Diagnostic and Statistical Manual of Mental Disorders, Fifth Edition (DSM-5) and International Classification of Sleep Disorders, third edition (ICSD-3) defined insomnia disorder. Moreover, they showed good concordance with measures of daytime dysfunction, as well as subjective and objective sleep [22]. The specific scores of 15 or more defined moderate-severe insomnia.

### Quality control for data collection

Each participants was assessed by scanning the Quick Response (QR) code of the questionnaires with internet-based electronic devices, and the questionnaires filling were guided by the researchers who had at least 2 years of psychological experiences. Before filling the questionnaires, the informed consent of the study was obtained. In order to assure the study quality, the questionnaires have set empty item reminders. There is a logical contradiction between the PHQ-9 items related to sleep and anxiety and the GAD-7 and ISI items. For example, participants who selected “nearly every day” at item “trouble falling/staying asleep, sleeping too much” of PHQ-9 selected “none” or “mild” at item “difficulty falling asleep” and/or “difficulty staying asleep” of ISI. This kinds of situation will receive a warning about logical contradiction between the questionnaires from online platform when the participants needed to reselected the items according to the real experiences. The three major questionnaires finished < 90 or > 1800 s was excluded in the analysis.

### Statistical analysis

IBM SPSS version 22.0 for Windows was used for all statistical tests. Proportions (%) was used to presented categorical variables, and mean ± standard deviation (x ± s) was used to present continuous variables. Independent-samples T test, Chi-square test were performed to assess the demographics, score of questionnaires, and severity of psychological problems in two groups. The odds ratio (OR) of Chi-square test were built to assess the association between score of questionnaires and demographic features. A value of two-tailed *p* < 0.05 was considered statistically significant.

## Results

### Sample recruitment procedure (Fig. 1)

**Fig. 1:**
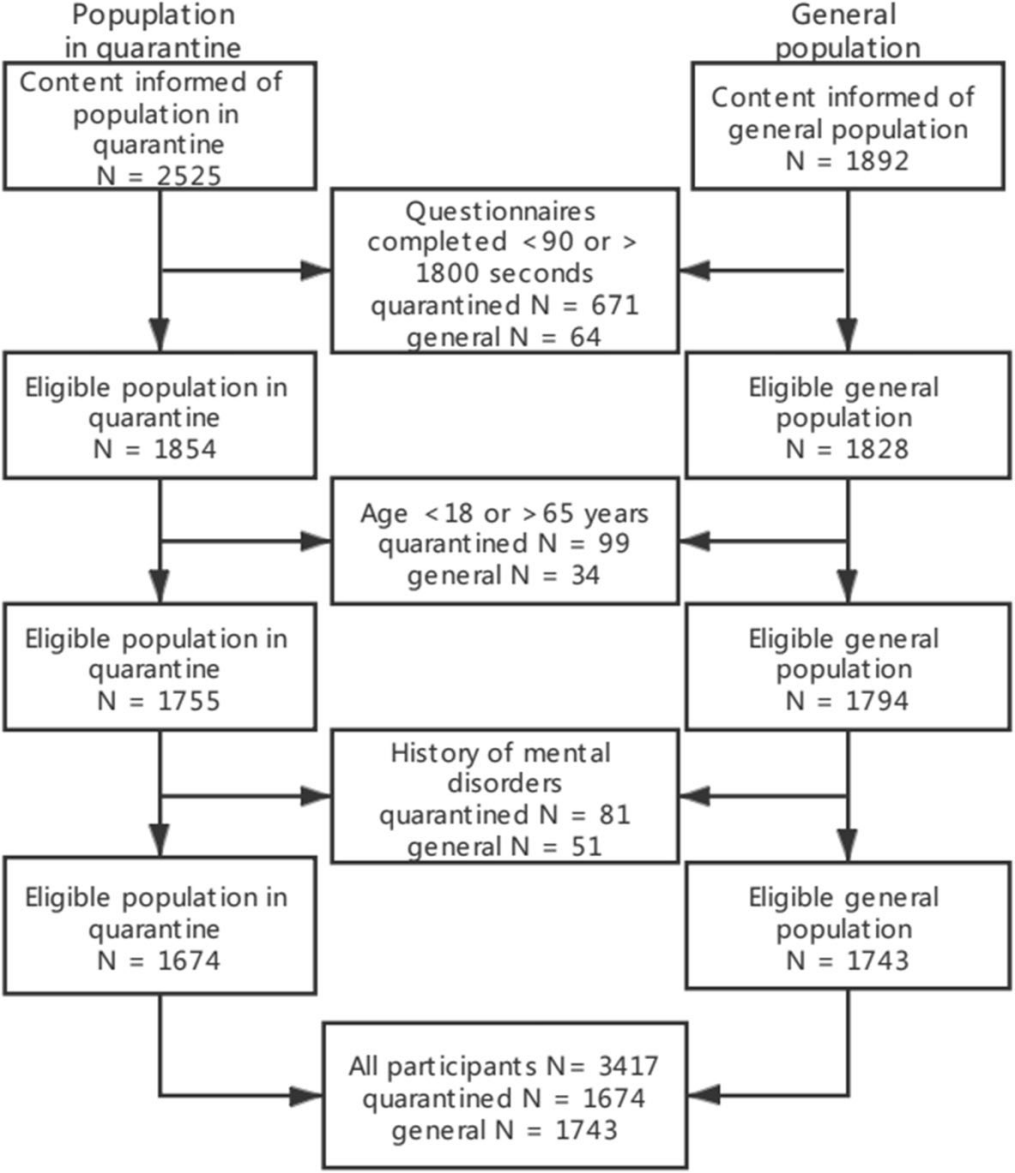
Flowchart of participants recruitment

The Fig. 1 shows the flowchart of sample recruitment process. A total of 2525 population in quarantine had given the content informed and completed the questionnaires. Among them, 1854 participants completed questionnaires between 90 and 1800 s. A total of 1755 met the criterion of age. Finally, 1674 participants were recruited with a response rate of 66.3%. We had screened 1892 general population from7th April to 15th June 2020 in the same region of Shenzhen, 1743 general population have given the informed consent and were matched the population in quarantine using age and sex with a ratio of about 1:1 (1743:1647).

### Demographics of general population and population in quarantine (Table 1)

Table 1 shows that a total of 1674 participants in quarantine as cases and 1743 general population participated as controls in this study. The mean age was 32.6 ± 9.3 years and 32.7 ± 10.7 years in cases and controls, respectively. There were no significant differences in the age, sex, education, marital status, household income, and any chronic medical disease between groups (*p* > 0.05). Population in quarantine had higher scores on PHQ-9, GAD-7, and ISI compared to controls (Cohen’ s d: 0.77, 0.55, and 0.43 respectively), suggesting that population in quarantine had higher risk of depressive, anxiety, and insomnia symptoms than controls.

**Table 1:** demographics and depressive, anxiety, and insomnia symptoms between general population and population in quarantine (*N* = 3417)

Demographics/Scales	Population in quarantine (*N* = 1674)	General population (*N* = 1743)	t/χ^2^	p	Effect sizes (Phi/Cohen’ s d)
Age, mean ± SD	32.6 ± 9.3	32.7 ± 10.7	0.15	0.88	0.00
Female, N (%)	834 (49.8%)	833 (47.8%)	1.49	0.24	0.02
Education (college or above), N (%)	1041 (62.2%)	1115 (64.0%)	1.68	0.28	0.02
Married, N (%)	880 (52.6%)	862 (49.5%)	3.31	0.07	0.03
Household income (> 7000 yuan / month)	1442 (86.1%)	1499 (86.0%)	0.01	0.91	0.00
Any chronic medical disease, N (%)	35 (2.1%)	43 (2.5%)	6.50	0.09	0.08
Hypertension	2 (0.1%)	10 (0.6%)	5.04	0.03	0.04
Diabetes	2 (0.1%)	5(0.3%)	1.18	0.45	0.02
Other chronic Physical conditions	31 (1.9%)	28 (1.6%)	0.30	0.58	0.01
PHQ-9, mean ± SD	6.1 ± 5.5	3.0 ± 3.7	−22.46	0.00	0.77
GAD-7, mean ± SD	4.2 ± 4.7	1.9 ± 3.7	−16.12	0.00	0.55
ISI, mean ± SD	5.5 ± 5.8	3.1 ± 5.0	−12.46	0.00	0.43

### Point prevalence and odds ratio of depression, anxiety, and insomnia between population in quarantine and general population based on the questionnaires’ cutoff points (Table 2)

Table 2 shows the point prevalence and odds ratio of depression, anxiety, and insomnia between two groups based on the questionnaires’ cutoff points. Currently, there were significant higher prevalence of depression (38.8% vs. 12.2%, *p* < 0.001), anxiety (27.0% vs. 11.2%, *p* < 0.001), and insomnia (27.5% vs. 5.5%, *p* < 0.001) among population in quarantine, when compared to controls. According to the results of Chi-square text (Table 2), population in quarantine showed significantly higher risks of depression (OR: 4.55, 95% CI: 3.82–5.41), anxiety (OR: 2.92, 95% CI: 2.43–3.51), and insomnia (OR: 2.40, 95% CI: 2.02–2.89), when compared to the general population.

**Table 2:** point prevalent and odds ratio (OR) of depression, anxiety, and insomnia of the participants between two groups based on the questionnaires

	Quarantine (*N* = 1647)	General (N = 1743)	P	OR	95% Confidence Interval	
					Lower	Upper
PHQ-9 ≥ 7, N (%)	649 (38.8%)	213 (12.2%)	0.00*	4.55	3.82	5.41
GAD-7 ≥ 7, N (%)	452 (27.0%)	196 (11.2%)	0.00*	2.92	2.43	3.51
ISI ≥ 8, N (%)	461 (27.5%)	238 (5.5%)	0.00*	2.40	2.02	2.89

Note: * *P*<0.05

### The severity of depression, anxiety, and insomnia between population in quarantine and general population based on the questionnaires’ scores (Fig. 2)

**Fig. 2:**
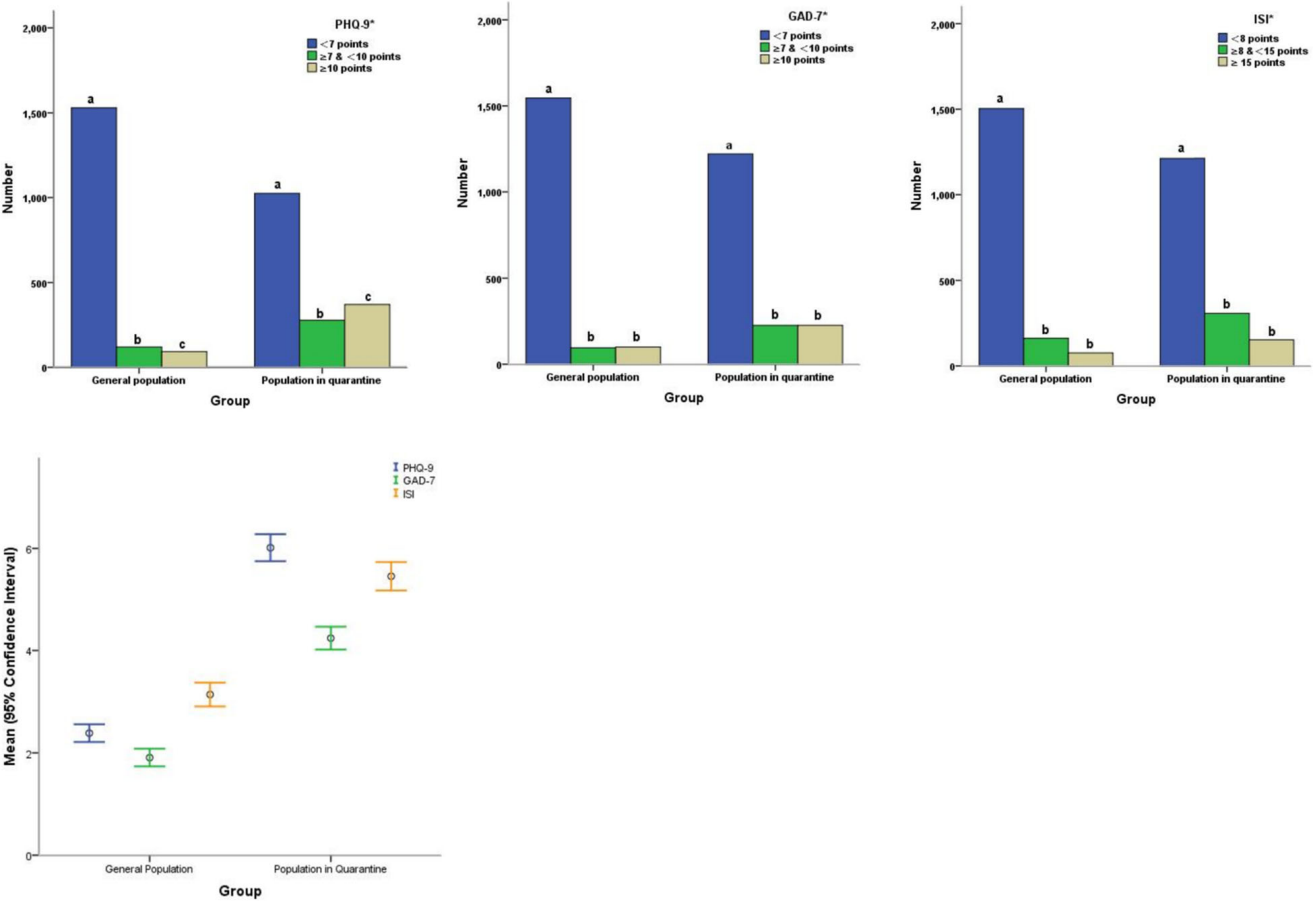
compared the severity of depression, anxiety and insomnia between two groups. The mean difference of PHQ-9, GAD-7, and ISI scores between two groups were 3.63 (95% CI: 3.31–3.94), 2.33 (95% CI: 2.05–2.62), and 2.31 (95%CI: 1.95–2.67), respectively

Figure 2 shows the severity of depression, anxiety, and insomnia between two groups based on the questionnaires’ scores. According to the result of Chi-square z-test, population in quarantine showed significantly higher severity of depression (22.2% vs. 5.3%, *p* < 0.001), when compared to controls. However, there was no significant difference in severity of anxiety and insomnia (*p* > 0.05). The mean difference of PHQ-9, GAD-7, and ISI scores between two groups were 3.63 (95% CI: 3.31–3.94), 2.33 (95% CI: 2.05–2.62), and 2.31 (95%CI: 1.95–2.67), respectively.

### The association between depressive, anxiety, insomnia symptoms and demography features among population in quarantine (Table 3)

According to the results of Chi-square test (Table 3), younger (OR: 1.74, 95% CI: 1.41–2.13), more educated (OR: 1.66, 95% CI: 1.35–2.04), non-married (OR: 1.82, 95% CI: 1.50–2.22), less household income (OR: 1.85, 95% CI: 1.40–2.44), and any chronic medical diseases (OR: 2.14, 95% CI: 1.09–4.21) population in quarantine showed higher risks of depression symptoms, when compared to the older, less educated, married, more household income, and undiagnosed any chronic medical diseases population in quarantine; Younger (OR: 1.56, 95% CI: 1.24–1.95), more educated (OR: 1.39, 95% CI: 1.11–1.74), non-married (OR: 1.58, 95% CI: 1.27–1.96), and less household income (OR: 1.58, 95% CI: 1.18–2.13) population in quarantine showed higher risks of anxiety symptoms, when compared to the older, less educated, married, and more household income in quarantine; Younger (OR: 1.39, 95% CI: 1.11–1.74), more educated (OR: 1.31, 95% CI: 1.05–1.65), non-married (OR: 1.71, 95% CI: 1.37–2.12), and any chronic medical diseases (OR: 2.01, 95% CI: 1.02–3.95) population in quarantine showed higher risks of insomnia symptoms, when compared to the older, less educated, married, and undiagnosed any chronic medical diseases population in quarantine. However, sex showed no significant association with mental health problems among population under quarantine. Household income and any chronic medical diseases showed no significant association with insomnia symptoms and anxiety symptoms respectively among population under quarantine.

**Table 3:** the association between depressive, anxiety, and insomnia symptoms and demography features among population under quarantine (N = 1647)

	PHQ-9		GAD-7		ISI	
	OR	95%CI	OR	95%CI	OR	95%CI
Age*	1.74	1.41–2.13	1.56	1.24–1.95	1.39	1.11–1.74
Sex	1.13	0.92–1.37	1.13	0.91–1.40	0.99	0.80–1.22
Education	1.66	1.35–2.04	1.39	1.11–1.74	1.31	1.05–1.65
Marital status	1.82	1.50–2.22	1.58	1.27–1.96	1.71	1.37–2.12
Household income	1.85	1.407–2.44	1.58	1.18–2.13	1.22	0.90–1.65
Any chronic medical diseases	2.14	1.09–4.21	1.08	0.52–2.27	2.01	1.02–3.95

Note: *: the cutoff age was defined with the mean age of the population in quarantine. The age < 33 years means “younger”. OR: odds ratio; 95%CI: 95% Confidence Interval

## Discussion

In this case-controlled study with a satisfactory sample size, we have proved our hypothesis that population in quarantine had a higher level of depressive, anxiety, and insomnia symptoms than their age-sex matched controls. Specifically, they were at a higher point prevalence of depression, anxiety, and insomnia, especially the severity of depression, when compared to controls. Younger, more education, non-married and lower household income showed higher risks of mental health problems. This study provided timely data on mental health problems among those populations in quarantine and raised the concerns of mental health problems for them.

There were a lot of articles reported a high burden of mental health problems among patients, informal caregivers, and healthcare providers who experienced quarantine or isolation [15]. The higher burden health problems among population under quarantine or isolation included depression, anxiety, and insomnia, which was similar compared to our studies. However, most of these studies were conducted in high-income and hospital settings that differed from our study setting [15]. One study conducted in southwestern China compared the quarantine affected group (they or their families/colleagues/classmates/neighbors had been quarantined) versus the quarantine unaffected group, and showed that the prevalence of anxiety and depression of the affected group are higher than of the unaffected group during the COVID-19 outbreak [16]. Similar results was also found in our case-controlled design which focused on population in quarantine and age-sex matched general population in the same region. Different from our study findings, the curve of searchers for suicidal ideation, anxiety, negative thoughts, and sleep disturbances in the United States presented a significant flattening after the implementation of stay-at-home orders, which suggested that the stay-at-home order had beneficial in reducing the level of anxiety about the risk of COVID-19 infection [23, 24].. But according to our study findings, the quarantine itself played a core role in the higher level of mental health problems among population in quarantine since the COVID-19 test of population in quarantine of our study were all negative, which means that they were uninfected. Similar to our study, Young population have higher risks of general psychological problems, while having a job and living with a partner are protective factors [25]. Our findings suggesting that more educated population had higher level of mental health problems was not consistent with previous study that lower education level was significantly associated with higher risk of psychiatric disorders [26, 27]. More educated population who had good knowledge and attitudes regarding COVID-19, but unsatisfactory preventive practices [28] might serve as explanation of our Findings.

The exact mechanisms underlying the higher risk of mental health problems among population in quarantine are largely unclear. Despite the findings of small association between demography features and mental health problems, the influence of the quarantined environment on mental health seems multifactorial and multidimensional. As pointed out by a recent review, the high-level stress state associated with the COVID-19 pandemic may lead to the elevated inflammatory level in the population under quarantine, which might cause mood and sleep disorders [29]. Tao et al. found that limited lighting and activities may associate with disturbances on circadian rhythm in a quarantined environment for 2 weeks, which might contribute to poor mental health outcomes [30]. It has been suggested that the stigma generated by quarantine is related to the mental and psychological problems [31], although stigma are not considered as traumatic event. As suggested by our findings, younger female population in quarantine with more education, non-married and lower income had more depressive, anxiety, and insomnia symptoms, which may also contribute to poor mental health outcomes among population in quarantine.

The strengths of the current study include a satisfactory sample size and moderate response rate, and well-matched controls for comparison. However, several limitations should be noted. Our study used self-reported assessment questionnaire, and lacked clinical interview by using a valid diagnostic tool for mental disorders. A lack of follow-up in our case-controlled study restricted inspection the longer-term impact of quarantine on mental health. Due to infectious control, the assessment was conducted through telephone and electronic devices rather than face-to-face guidance, which may lead to the underestimation of mental health problems. We will consider using valid clinical diagnostic interview tools to establish the mental health problems and conduct baseline and follow-up face-to-face assessment in future study.

In conclusion, the implementation of centralized 2 weeks’ quarantine measures affected the mental health of population under quarantine. The results of our study indicate that depressive, anxiety, and insomnia symptoms were more common and severe among the population under 2 weeks’ quarantine, when compared to the general population. Specifically, younger population in quarantine with more education, non-married and lower income were at higher risks of mental health problems. Our findings suggests that more necessitates multipronged interventions including strengthening the psychological assessment before 2 weeks’ quarantine and providing mental health consulting in population under quarantine. The government should implement the home isolation policy or shortening the duration of quarantine while the population had unstable mental health status according to the psychiatric’ assessment or interview are needed to promote mental health among high-risk populations including that under 2 weeks’ quarantine. Mental health professionals shall pay more attention to the mental health problems for those population in quarantine.

## Supplementary Information

**Additional file 1.**

## Data Availability

All data requests should be the corresponding author (Dr. Weidong Song) for consideration. Considering the raw data contained the information of name, address, cell phone number, and ID number, the government do not allowed to share the original date.
