# Supplementary material for: Depressive, anxiety, and insomnia symptoms between population in quarantine and general population during the COVID-19 pandemic: a case-controlled study
Source: BMC Psychiatry. 2021 Feb 16;21:99. doi: 10.1186/s12888-021-03108-2 (PMC7884871; doi:10.1186/s12888-021-03108-2)
Supplement: Supplementary file 1 — Additional file 1. [file 12888_2021_3108_MOESM1_ESM.docx]

Depressive, anxiety, and insomnia symptoms between population in quarantine and public population during the COVID-19 pandemic: a case-controlled study

Please read the consent informed carefully, and decided whether or not to participant our study!

In order to determine the impact of mental and psychological symptoms among individuals during COVID-19 pandemic, we conduct this convey study which was approved by The Control and Prevention Commend Office of COVID-19 Pandemic in Longgang district, Shenzhen (Document NO.: [2020]90). If you agree to participant and content to public your assessment data, please select “agree” and you will start your assessment.

□ agree □ do not agree

Time:

Medical observations check point:

Demographic
1. Name

_________________________________

2. Age

_________________________________

3. Sex (select)

| □ male |
| --- |
| □ female |

4. Telephone number

_________________________________

5. Name of Relatives or friends

_________________________________

6. Telephone number of relatives or friends

_________________________________

7. Education (select)

| □ college or above |
| --- |
| □ college below |

8. Marital status (select)

| □ married |
| --- |
| □ others (including unmarried, divorced, and widowed et al.) |

9. Household income (select)

| □ > 7000 yuan / month |
| --- |
| □ ≤ 7000 yuan / month |

10. Medical history (select and fill)

| □ mental disorders (including suicide thought or behavior, major stress event, and substance abuse) |
| --- |
|  |
| □ other chronic medical disease  _________________________________    11. Room number:  12. Current status (select)   \| □ in - quarantine \| \| --- \| \| □ out - quarantine \| |

Patient Health Questionnaire -9 (PHQ-9)
Over the last 2 weeks, how often have you been bothered by any of the following problems?

|  | Not at all | Several days | More than half the days | Nearly every day |
| --- | --- | --- | --- | --- |
| Little interest or pleasure in doing things | □ | □ | □ | □ |
| Feeling down, depressed, or hopeless | □ | □ | □ | □ |
| Trouble falling/staying asleep, sleeping too much | □ | □ | □ | □ |
| Feeling tired or having little energy | □ | □ | □ | □ |
| Poor appetite or overeating | □ | □ | □ | □ |
| Feel bad about yourself or that you are a failure or have let yourself or your family dawn | □ | □ | □ | □ |
| Trouble concentrating on things, such as reading the new paper or watching television | □ | □ | □ | □ |
| Moving or speaking so slowly that other people could have noticed. Or the opposite, being so fidgety or restless that you have been moving around a lot more than usual | □ | □ | □ | □ |
| Thoughts that you would be better off dead or hurting yourself in some way | □ | □ | □ | □ |

Generalized Anxiety Scale (GAD-7)
Over the last 2 weeks, how often have you been bothered by the following problems?

|  | Not at all sure | Several days | Over half the days | Nearly every day |
| --- | --- | --- | --- | --- |
| Feeling nervous, anxious, or on edge | □ | □ | □ | □ |
| Not being able to stop or control worrying | □ | □ | □ | □ |
| Worrying too much about different things | □ | □ | □ | □ |
| Trouble relaxing | □ | □ | □ | □ |
| Being so restless that it’s hard to sit still | □ | □ | □ | □ |
| Becoming easily annoyed or irritable | □ | □ | □ | □ |
| Feeling afraid as if something awful might happen | □ | □ | □ | □ |

Insomnia Severity Index (ISI)
For each question below, please select the most accurately to your sleep patterns in the last 2 weeks.

|  | None | Mild | Moderate | Severe | Very Severe |
| --- | --- | --- | --- | --- | --- |
| Difficulty falling asleep | □ | □ | □ | □ | □ |
| Difficulty staying asleep | □ | □ | □ | □ | □ |
| Problem waking up too early in the morning | □ | □ | □ | □ | □ |
|  | Very Satisfied | Satisfied | Neutral | Dissatisfied | Very Dissatisfied |
| How SATISFIED/dissatisfied are you with your current sleep pattern? | ○ | ○ | ○ | ○ | ○ |
|  | Not at all | A Little | Somewhat | Very | Very Much |
| To what extent do you consider your sleep problem to INTERFERE with your daily functioning (e.g., daytime fatigue, ability to function at work/daily chores, concentration, memory, mood) | □ | □ | □ | □ | □ |
| How NOTICEABLE to others do you think your sleeping problem is in terms of impairing the quality of your life? | □ | □ | □ | □ | □ |
| How WORRIED / distressed are you about your current sleep problem? | □ | □ | □ | □ | □ |
